# Transaxillar Impella Implantation: Learning Curve Analysis and the Role of Mentorship in Accelerating Proficiency

**DOI:** 10.3390/jcm15135154

**Published:** 2026-07-02

**Authors:** Serena Boeddu, Marcin P. Szczechowicz, Kálmán Benke, Fabio Abbondanza, Anna Hoffmeister, Viktor Banhegyi, Givi Damenija, Gábor Szabó, Gábor Veres

**Affiliations:** 1 Department of Cardiac Surgery, University Hospital Halle, 06120 Halle (Saale), Germanyfabio.abbondanza@uk-halle.de (F.A.); anna.hoffmeister@uk-halle.de (A.H.); gabor.szabo@uk-halle.de (G.S.);; 2 Department of Cardiac Surgery, Clinical Provincial Hospital No. 2 of Saint Queen Jadwiga, 35-301 Rzeszów, Poland; 3 Department of Cardiac Surgery, Heart and Vascular Center, Semmelweis University, 1094 Budapest, Hungary; gdamenija@gmail.com; 4 Department of Cardiac Surgery, University Hospital Heidelberg, 69120 Heidelberg, Germany

**Keywords:** Impella, mechanical circulatory support, transaxillar approach, learning curve, mentorship, radiation exposure

## Abstract

**Objectives:** Transaxillar Impella 5.0/5.5 implantation is a hybrid surgical and fluoroscopy-guided procedure. We evaluated the learning curve using radiation exposure as a marker of procedural efficiency and assessed whether structured mentorship accelerates procedural proficiency. **Methods:** This retrospective single-center study included consecutive transaxillar Impella 5.0/5.5 implantation attempts by two surgeons. Surgeon A adopted the technique independently, whereas Surgeon B was trained under direct proctorship. The primary endpoint was radiation exposure (dose–area product), and the secondary endpoint was fluoroscopy time. Temporal trends were analyzed by regression, and CUSUM plots were generated. **Results:** Of 104 procedures, 14 were excluded (12 transaortic, 2 unsuccessful). Ninety procedures were analyzed (74 Surgeon A, 16 Surgeon B). In Surgeon A, radiation exposure decreased significantly with increasing case number. In Surgeon B, no significant association between case number and radiation exposure was observed. Fluoroscopy time was not associated with case number in either group. CUSUM analysis suggested an early increase followed by stabilization in Surgeon A, whereas no clear pattern was observed in Surgeon B. The between-surgeon interaction was not statistically significant. ECMELLA configuration was the only independent predictor of increased radiation exposure, whereas device type, surgery type, and patient age were not significant predictors. **Conclusions:** Transaxillar Impella implantation appears to have a measurable early learning phase. Structured mentorship may attenuate the early learning phase, although this finding remains exploratory.

## 1. Introduction

Temporary mechanical circulatory support has become an important component of the management of advanced heart failure, life-threatening arrhythmias, cardiogenic shock and selected high-risk cardiac interventions [1,2,3,4]. The Impella microaxial flow pump provides direct left ventricular unloading with rapid hemodynamic stabilization and is increasingly used across a broad spectrum of indications, including perioperative support in high-risk cardiac surgery and bridging to more durable therapies [5,6].

Surgical transaxillar implantation offers several practical advantages over other strategies, including facilitation of prolonged support, earlier mobilization and reduced risk of lower-extremity complications [5,6,7]. However, it is technically demanding because it combines surgical arterial exposure with fluoroscopic wire, catheter and device manipulation in a hybrid procedural workflow [7]. As a result, the procedure may be particularly susceptible to operator learning effects [8,9].

Learning curves have long been recognized in surgery and have been described in a wide range of cardiovascular interventions [8,10]. In transcatheter procedures such as transcatheter aortic valve implantation, transcatheter edge-to-edge mitral repair and other structural heart interventions, growing operator experience has been associated with improved procedural efficiency and, in some settings, better clinical outcomes [9,10,11,12]. Several complementary methods have been used to evaluate procedural learning, including regression-based temporal analyses and cumulative sum (CUSUM) techniques, the latter being particularly useful for visualizing changes in performance over sequential cases [13].

Radiation exposure is a relevant marker of procedural efficiency in fluoroscopy-guided interventions [14,15]. Beyond occupational safety considerations, radiation dose reflects procedural planning, imaging strategy, device manipulation and the need for repeated corrective maneuvers [16,17]. Studies in structural heart interventions have shown that increasing operator experience may be accompanied by reductions in radiation burden even when other procedural metrics change less markedly [12,18].

While operator learning curves have been investigated in several cardiovascular procedures, the effect of structured mentorship on procedural adoption remains less well defined [19,20,21]. Proctorship has been advocated during the introduction of technically demanding techniques, including structural heart interventions and minimally invasive cardiac surgery, but direct evidence comparing mentored and independently acquired learning trajectories remains limited [19,21].

Despite the growing use of transaxillar Impella implantation, data specifically addressing the learning curve of this hybrid technique remain scarce [5,6,7]. The present study therefore evaluated sequential changes in radiation exposure during transaxillar Impella 5.0/5.5 implantation and explored whether prior mentorship was associated with attenuation of the early learning phase.

## 2. Patients and Methods

### 2.1. Study Design and Population

This retrospective two-surgeon study evaluated the learning curves associated with transaxillar implantation of Impella 5.0 and 5.5 microaxial flow pumps (Abiomed Europe GmbH, Aachen, Germany) at a single academic cardiac surgery center (University Hospital Halle, Germany).

Surgeon A adopted the technique independently, performing the first implantation in February 2021. Surgeon B subsequently began performing these procedures in 2024 under direct proctorship from Surgeon A and using the same standardized surgical technique. Proctorship consisted of the physical presence of the experienced operator in the operating room with guidance during device navigation and fluoroscopic positioning.

All consecutive implantation attempts were screened. Transaortic implantations were excluded because this approach involves a fundamentally different access route and fluoroscopic strategy. Cases in which device implantation was attempted but not successfully completed, were not included in the primary analysis of procedural radiation burden, but they were recorded separately and reported descriptively as procedural failures. No exclusion criteria were applied regarding patient characteristics, device type (Impella 5.0 vs. 5.5), clinical indication, or implantation setting (intraoperative, postoperative, or standalone), as the implantation technique remained unchanged throughout the study period. The primary analytic cohort therefore consisted of successful transaxillar implantations. Procedures were grouped according to the primary operating surgeon.

### 2.2. Ethics

The study was approved by the Ethics Committee of the Martin Luther University Halle-Wittenberg (approval number: 2026-054). The requirement for individual written informed consent was waived by the Ethics Committee in view of the retrospective study design and the use of fully anonymized registry data.

### 2.3. Data Collection

Data were extracted from a prospectively maintained institutional registry. Radiation exposure was expressed as dose–area product (DAP, cGy·cm^2^) as recorded by the integrated fluoroscopy dosimeter, and fluoroscopy time was recorded in minutes. The implantation setting was categorized as intraoperative (during concomitant cardiac surgery), postoperative (following prior cardiac surgery for postcardiotomy hemodynamic compromise), or standalone (preoperative mechanical circulatory support without concomitant cardiac surgery). ECMELLA configuration (Impella implantation in the presence of extracorporeal membrane oxygenation) was documented prospectively, distinguishing between primary ECMELLA implantation and escalation from pre-existing ECMO support. Concomitant cardiac procedures were categorized as isolated CABG, valve surgery with or without CABG, or other procedures.

### 2.4. Study Endpoints

The primary endpoint was procedural radiation exposure, assessed by dose-area product (DAP). The secondary endpoint was fluoroscopy time. The principal study aim was to assess temporal change in these metrics with increasing procedural experience.

A secondary aim was to explore whether the learning pattern differed between the surgeon who established the program and the subsequently mentored surgeon.

DAP was selected as the primary endpoint because it integrates fluoroscopy duration, field size, and acquisition frequency into a single automatically recorded, observer-independent metric. In fluoroscopy- guided procedures, DAP reflects not only imaging duration but also decisions regarding magnification, projection selection, and the frequency of repeated confirmatory acquisitions, making it a more sensitive marker of procedural fluency than fluoroscopy time alone, as demonstrated in structural heart interventions [14,16].

### 2.5. Definition of Learning Curves

Learning curves were assessed using the chronological case number of each surgeon as the measure of accumulating experience. For the primary analyses, case number was treated as a continuous variable. This approach was chosen to preserve statistical efficiency and avoid arbitrary categorization of experience.

Because procedural learning may be non-linear, graphical assessment of temporal trends was performed in parallel with formal regression analyses. In addition, cumulative sum (CUSUM) plots were generated for DAP as exploratory visual tools to illustrate temporal changes in radiation burden across consecutive cases. For each surgeon, CUSUM was calculated as the running sum of deviations of individual DAP values from the surgeon-specific mean DAP of the analyzed series. Changes in curve direction were interpreted descriptively as possible indicators of transition from an early learning phase to greater procedural stability. CUSUM analyses were not used as confirmatory tests of a specific proficiency threshold.

### 2.6. Statistical Analysis

Statistical analyses were performed using RStudio Pro 2026.01.1 Build 403.pro11.

Given the limited sample size and the exploratory nature of the study, the statistical strategy was intentionally restricted to a small number of clinically relevant analyses.

Continuous variables are reported as median with interquartile range or mean ± standard deviation, as appropriate according to distribution. Categorical variables are presented as counts and percentages.

DAP showed a right-skewed distribution and was therefore analyzed primarily on the logarithmic scale. The association between experience and radiation exposure was assessed using linear regression with log-transformed DAP as the dependent variable and case number as the main independent variable. Fluoroscopy time was analyzed analogously as a secondary endpoint. For better visualization, fitted trend lines were displayed graphically over the observed data.

The principal learning-curve analysis was performed for Surgeon A, who contributed the larger and methodologically more interpretable case series. For Surgeon B, analyses were considered exploratory because of the smaller number of cases and the resulting limited power to detect temporal trends reliably.

To explore whether learning patterns differed between surgeons, an additional regression model was fitted in the overall cohort including surgeon, case number, and a surgeon × case number interaction term.

A limited adjusted analysis was performed in the overall cohort including case number, surgeon, age, ECMELLA use, heart surgery, and Impella platform. Age was included as a continuous covariate. These analyses were intended to account for major procedural and patient-related factors expected to influence radiation burden, while avoiding excessive model complexity.

Regression assumptions were assessed by visual inspection of residual plots. Two observations exceeding the predefined outlier threshold were excluded in the sensitivity analysis, both from Surgeon A.

### 2.7. Exploratory Subgroup Analyses

Exploratory subgroup analyses were restricted to clinically relevant procedural factors expected to influence radiation burden, namely ECMELLA configuration, heart surgery, and Impella platform. Because of the limited sample size, particularly in Surgeon B, subgroup analyses were restricted to Surgeon A and were interpreted descriptively only.

### 2.8. Handling of Outliers

Extreme radiation values were not excluded from the primary analysis, as these cases may represent genuine procedural complexity rather than measurement error and may therefore form part of the learning process itself. Exclusion of such observations might attenuate the early learning phase artificially. Instead, a sensitivity analysis was performed in which extreme observations were excluded according to a uniform predefined rule.

Description and results. Extreme values were defined as observations exceeding Q3 + 3 × IQR for Surgeon A and Q3 + 1.5 × IQR for Surgeon B, applied separately per surgeon. Two observations were excluded in the sensitivity analysis, both from Surgeon A.

## 3. Results

### 3.1. Study Cohort

A total of 104 consecutive Impella implantation attempts were identified during the study period. After exclusion of 12 transaortic procedures, 92 dose–area product implantation attempts remained. Of these, 2 cases were classified as implantation failures and were therefore excluded from the primary endpoint analysis of successful procedures. However, we retained them in the cohort description.

Both unsuccessful attempts were aborted due to unsuitable vascular anatomy identified intraoperatively, and therefore do not represent operator-dependent procedural failures.

**Figure 1:**
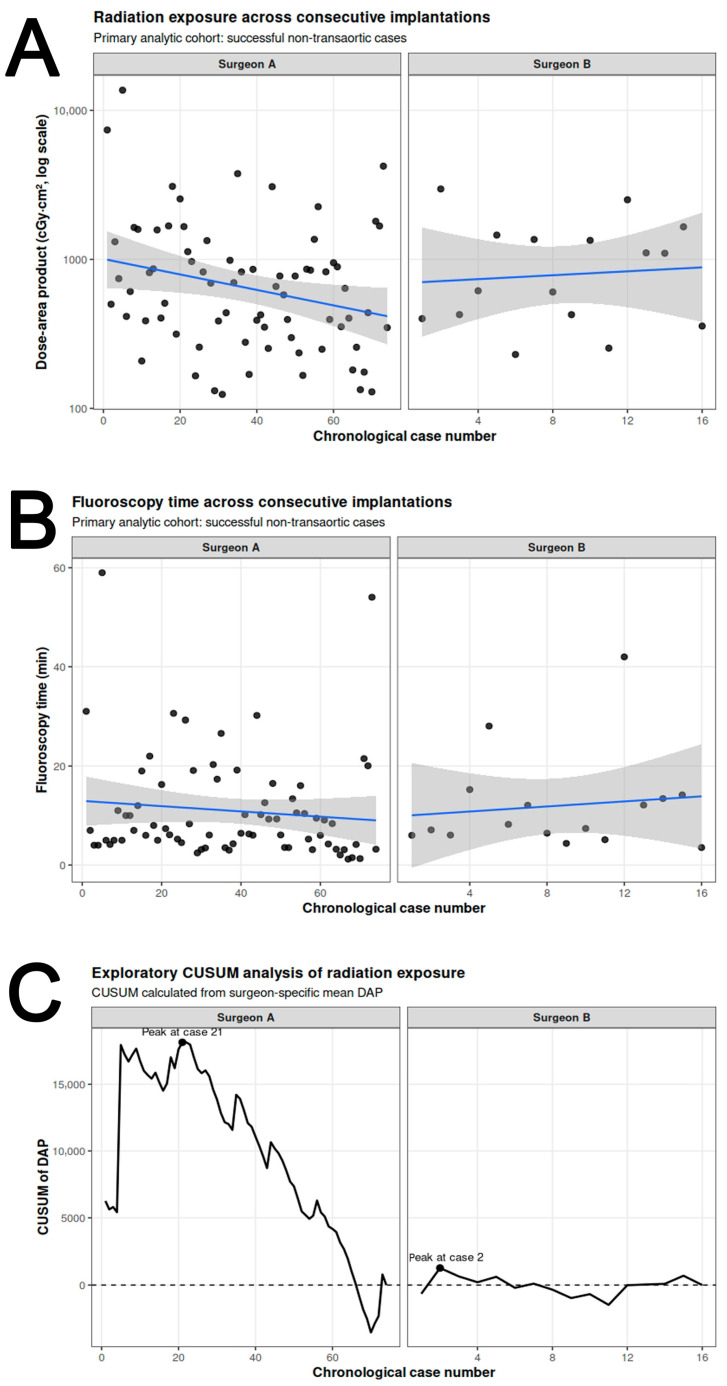
(**A**): Radiation exposure across consecutive dose–area producty Impella implantations.; (**B**): Fluoroscopy time across consecutive dose–area producty Impella implantations.; (**C**): Exploratory cumulative sum (CUSUM) analysis of radiation exposure.

The characteristics of included patients are described in Table 1. The final analytic cohort comprised 90 successful dose–area product implantations, including 74 procedures performed by Surgeon A and 16 procedures performed by Surgeon B (Figure 1, Table 2).

### 3.2. Patient and Procedural Characteristics

Impella 5.5 was used in most procedures, whereas Impella 5.0 was largely confined to the earlier phase of program implementation. ECMELLA configuration was present in a substantial proportion of cases in both surgeon groups (Table 1). The distribution of concomitant procedures is shown in Table 3, with standalone Impella implantation, CABG-only procedures and valve-associated procedures accounting for the majority of cases.

The presence of ECMO does not alter the technical steps of transaxillar Impella implantation itself; the higher radiation exposure observed in ECMELLA cases reflects the greater hemodynamic instability of these patients rather than a fundamentally different procedural technique.

### 3.3. Radiation Exposure over Time

Main procedure-related results are presented in Table 2. Radiation exposure plotted against chronological case number suggested a progressive decline in DAP over time in Surgeon A, whereas no similarly consistent pattern was apparent in Surgeon B (Figure 1A).

In the primary regression analysis, log-transformed DAP decreased significantly with increasing case number in Surgeon A, consistent with a learning-related reduction in procedural radiation burden. This corresponds to an approximate 1.2% reduction in DAP per additional procedure, or a cumulative reduction of approximately 21% over the first 20 cases. In contrast, no significant temporal association between case number and log-transformed DAP was observed in Surgeon B. The corresponding regression estimates, confidence intervals and *p*-values are presented in Table 4.

### 3.4. Fluoroscopy Time over Time

Fluoroscopy time plotted against chronological case number showed a directionally downward pattern in Surgeon A, whereas no clear temporal pattern was evident in Surgeon B (Figure 1B). However, compared with DAP, the temporal signal for fluoroscopy time was less pronounced. In the secondary regression analysis, no significant association between case number and fluoroscopy time was observed in both surgeons’ groups (Table 4).

### 3.5. Exploratory CUSUM Analysis

Exploratory CUSUM analysis of DAP showed different temporal patterns between the two operators (Figure 1C). For Surgeon A, the curve initially rose and then gradually changed direction, with the maximum cumulative deviation observed at approximately case 21, after which the trajectory became predominantly downward. This pattern was consistent with an early phase of higher radiation burden followed by greater procedural stability.

For Surgeon B, the CUSUM curve demonstrates different pattern. Its trajectory fluctuated across the small series without a stable direction (Figure 1C).

### 3.6. Comparison Between Surgeons

When all analyzable cases were considered together, overall procedural radiation exposure and fluoroscopy time were broadly comparable between surgeons descriptively (Table 2).

To explore whether learning patterns differed between surgeons, an interaction model including case number, surgeon, and a case number × surgeon interaction term was fitted. Although Surgeon A showed a significant inverse association between case number and DAP, the between-surgeon interaction was not statistically significant, indicating that a differential learning trajectory between surgeons could not be formally confirmed in this dataset. No significant between-surgeon interaction was observed for fluoroscopy time either (Table 4).

### 3.7. Adjusted Analyses

In adjusted regression analyses including case number, surgeon, age, ECMELLA use, heart surgery, and Impella platform, the association between case number and radiation burden was attenuated. Procedural complexity variables, particularly ECMELLA configuration, contributed to higher radiation exposure, whereas no independent between-surgeon difference in temporal trend was demonstrated.

In the adjusted model, ECMELLA configuration was independently associated with increased radiation exposure (*p* = 0.013), whereas device type (*p* = 0.41), surgery type (*p* = 0.055), and patient age (*p* = 0.134) were not significant independent predictors.

Adjusted analyses for DAP and fluoroscopy time are summarized in Supplementary Table S1.

### 3.8. Sensitivity Analyses

Sensitivity analyses excluding extreme radiation values according to a uniform predefined rule yielded findings that were directionally consistent with the primary analyses. Two observations were excluded based on the predefined criterion, both from Surgeon A. The inverse temporal pattern in DAP for Surgeon A remained visible after exclusion of extreme values, whereas no consistent temporal decline was observed in Surgeon B (Supplementary Figure S1). Similarly, exploratory CUSUM plots after exclusion of extreme values showed no material change in the overall interpretation of temporal patterns (Supplementary Figure S2).

Taken together, these sensitivity analyses did not materially alter the interpretation of the study and therefore supported the robustness of the primary findings.

### 3.9. Subgroup Analyses

Exploratory subgroup analyses restricted to Surgeon A are summarized in Supplementary Table S2. Radiation exposure was numerically higher in ECMELLA cases and in procedures performed in conjunction with heart surgery. The overall temporal pattern of decreasing radiation burden with increasing case number remained directionally similar across these procedural subsets. Procedures performed with Impella 5.5 showed lower radiation exposure than those performed with Impella 5.0, although this finding should be interpreted cautiously because device adoption and program maturation occurred over the same time period.

Subgroup plots for Surgeon A are shown in Supplementary Figures S3–S5, illustrating that the main temporal pattern remained directionally consistent across ECMELLA, concomitant procedures, and Impella-platform subsets.

## 4. Discussion

The present study demonstrates that transaxillar Impella 5.0/5.5 implantation is associated with a measurable learning curve when the technique is adopted independently. Radiation exposure decreased progressively with increasing case experience, and CUSUM analysis indicated procedural stabilization after approximately 20 cases. In contrast, no such pattern was observed in the mentored operator. These findings suggest that structured proctorship may attenuate the early learning phase of this hybrid surgical–fluoroscopic procedure.

The observed reduction in radiation exposure is likely multifactorial. Transaxillar Impella implantation involves a sequence of coordinated steps, including surgical arterial exposure, graft anastomosis, guidewire and catheter manipulation, and fluoroscopic confirmation of device positioning [5,6,7]. With increasing experience, these steps become more streamlined, and coordination between the surgical and imaging components improves. This is likely to reduce unnecessary imaging and repeated corrective maneuvers, resulting in lower overall radiation exposure.

Compared with other complex cardiovascular procedures, the learning phase observed in this study appears relatively short. Published data suggest that proficiency in TAVI and minimally invasive cardiac surgery may require substantially higher case numbers [9,11,13].

Direct comparisons with TAVI and minimally invasive cardiac surgery learning-curve literature should be interpreted with caution, as endpoint definitions and procedural complexity differ substantially between studies.

The findings in the mentored cohort should be interpreted cautiously. The absence of a detectable early inefficiency phase may reflect effective transfer of procedural knowledge through structured proctorship. However, given the small sample size and the absence of a statistically significant interaction between operators, the present study does not demonstrate elimination of the learning curve. Rather, it suggests that no clear early increase in radiation exposure was observed under mentored conditions.

This interpretation is consistent with the broader experience in structural heart interventions, where proctoring is widely used during the introduction of new techniques. Previous reports have shown that supervised adoption is feasible and supports safe implementation, although quantitative data on its effect on learning-curve dynamics remain limited [19,20,21,22,23,24]. The present study adds to this field by suggesting that mentorship may reduce the early procedural inefficiency associated with transaxillar Impella implantation.

Subgroup analyses provide additional clinical context. Radiation exposure was higher in ECMELLA cases, which is consistent with the increased hemodynamic and procedural complexity of these patients [3,6]. In contrast, device type and surgical context were not independent predictors of radiation burden, suggesting that the observed learning effect is not primarily driven by case mix. A trend toward higher radiation exposure in older patients was also observed, likely reflecting vascular characteristics such as calcification and tortuosity, although this did not reach statistical significance.

Importantly, the overall radiation burden remained low throughout the study. Even during the early learning phase, exposure was within the range reported for routine device implantation and substantially lower than that described for TAVI or PCI [16,18]. These findings indicate that radiation safety concerns should not limit the adoption of transaxillar Impella programs.

This study has several limitations. It represents a single-center experience with an imbalance between operators, limiting statistical power for comparative analyses. The use of radiation exposure as a surrogate for procedural efficiency does not capture clinical outcomes, which are primarily influenced by patient-related factors and would require larger, risk-adjusted analyses. CUSUM analysis was applied as an exploratory tool, and the identified threshold should not be interpreted as a definitive proficiency cut-off. In addition, temporal improvements in team performance cannot be fully separated from individual operator learning.

The comparison between surgeons was considered exploratory, as the non-significant interaction term (*p* = 0.595) and the small size of the mentored cohort (*n* = 16) limit the ability to draw definitive conclusions regarding the influence of structured proctorship on learning-curve characteristics.

Furthermore, the two surgical series were not contemporaneous, and concurrent improvements in institutional team coordination and device generation may have differentially influenced the results, representing an inherent limitation of any non-randomized two-surgeon comparison.

## 5. Conclusions

To our knowledge, this is the first study to formally evaluate the learning curve of transaxillar Impella 5.0/5.5 implantation as a hybrid surgical–fluoroscopic procedure. Despite these limitations, the findings have practical implications. The exploratory CUSUM analysis identified an inflection point at approximately case 20, which may serve as a preliminary reference for program planning but should not be interpreted as a validated proficiency threshold. The results support the use of structured proctorship during program initiation and identify ECMELLA configuration as a relevant source of procedural complexity. Finally, the consistently low radiation burden supports the broader implementation of transaxillar Impella implantation without major occupational safety concerns.

## Figures and Tables

**Table 1:** Patient and procedural characteristics.

	Surgeon A (*n* = 74)	Surgeon B (*n* = 16)
Age [years]	63.5 [57.2–70.8]	61.5 [57.2–68.5]
Female sex	12 (16.2%)	2 (12.5%)
Impella 5.5	59 (79.7%)	15 (93.8%)
ECMELLA	30 (40.5%)	7 (43.8%)
Bridge to LVAD	8 (10.8%)	2 (12.5%)
During heart surgery	42 (56.8%)	8 (50%)
During ECLS explantation	3 (4.1%)	1 (6.2%)

Abbreviations: ECMELLA—ECMO with Impella, LVAD—left ventricular assist device, ECMO—extracorporeal membrane oxygenation.

**Table 2:** Main descriptive results.

	Surgeon A	Surgeon B
Successful dose–area product implantations	74	16
Abandoned (unsuitable vascular anatomy)	1	1
Dose-area product [cGy·cm^2^]	648.4 [349.0–1090.8]	857.2 [419.2–1383.7]
Fluoroscopy time [min]	7.0 [4.2–13.2]	7.8 [6.0–13.6]

**Table 3:** Type of concomitant cardiac surgery among patients undergoing Impella implantation during cardiac surgery.

	Surgeon A (*n* = 74)	Surgeon B (*n* = 16)
Standalone Impella	7 (9.5%)	2 (12.5%)
CABG only	21 (28.4%)	6 (37.5%)
Aortic valve surgery only	6 (8.1%)	1 (6.2%)
Valve and aortic procedure	1 (1.4%)	1 (6.2%)
Valve and CABG	10 (13.5%)	0
Valve and aortic procedure and CABG	1 (1.4%)	0
Multiple valve procedure	1 (1.4%)	0
VSD repair	2 (2.7%)	0

Abbreviations: CABG: coronary artery bypass grafting, VSD—ventricular septal defect.

**Table 4:** Main trend analyses.

Outcome	Analysis	Estimate (95% CI)	*p*-Value
log(DAP)	Surgeon A	−0.012 (−0.022 to −0.002)	0.024
log(DAP)	Surgeon B	0.015 (−0.081 to 0.111)	0.740
log(DAP)	Between-surgeon interaction	0.027 (−0.074 to 0.128)	0.595
Fluoroscopy time	Surgeon A	−0.053 (−0.170 to 0.064)	0.367
Fluoroscopy time	Surgeon B	0.255 (−0.946 to 1.457)	0.655
Fluoroscopy time	Between-surgeon interaction	0.309 (−0.851 to 1.468)	0.598

Abbreviations: DAP—dose–area product.

## Data Availability

The data underlying this article are available in the article and its online Supplementary Material. Additional anonymized data can be made available upon reasonable request to the corresponding author.

## Supplementary Materials

The following supporting information can be downloaded at: https://www.mdpi.com/article/10.3390/jcm15135154/s1, Supplementary Figure S1: Sensitivity analysis of radiation exposure after exclusion of extreme values. Supplementary Figure S2: Sensitivity CUSUM analysis after exclusion of extreme radiation values. Supplementary Figure S3: Exploratory subgroup analysis by ECMELLA configuration in Surgeon A. Supplementary Figure S4: Exploratory subgroup analysis by heart surgery status in Surgeon A. Supplementary Figure S5: Exploratory subgroup analysis by Impella platform in Surgeon A. Supplementary Table S1: Adjusted regression analyses. Supplementary Table S2: Exploratory subgroup results in Surgeon A.
