# Supplementary material for: Transaxillar Impella Implantation: Learning Curve Analysis and the Role of Mentorship in Accelerating Proficiency
Source: J Clin Med. 2026 Jul 2;15(13):5154. doi: 10.3390/jcm15135154 (PMC13363602; doi:10.3390/jcm15135154)
Supplement: Supplementary file 1 [file jcm-15-05154-s001.zip › jcm-4277785-supplementary.pdf]

Supplementary Figures

Supplementary Figure S1. Sensitivity analysis of radiation exposure after exclusion of extreme values.

Supplementary Figure S2. Sensitivity CUSUM analysis after exclusion of extreme radiation values.

Supplementary Figure S3. Exploratory subgroup analysis by ECMELLA configuration in Surgeon A.

Supplementary Figure S4. Exploratory subgroup analysis by heart surgery status in Surgeon A.

Supplementary Figure S5. Exploratory subgroup analysis by Impella platform in Surgeon A.

Supplementary Figure S1. Sensitivity analysis of radiation exposure after exclusion of extreme values.

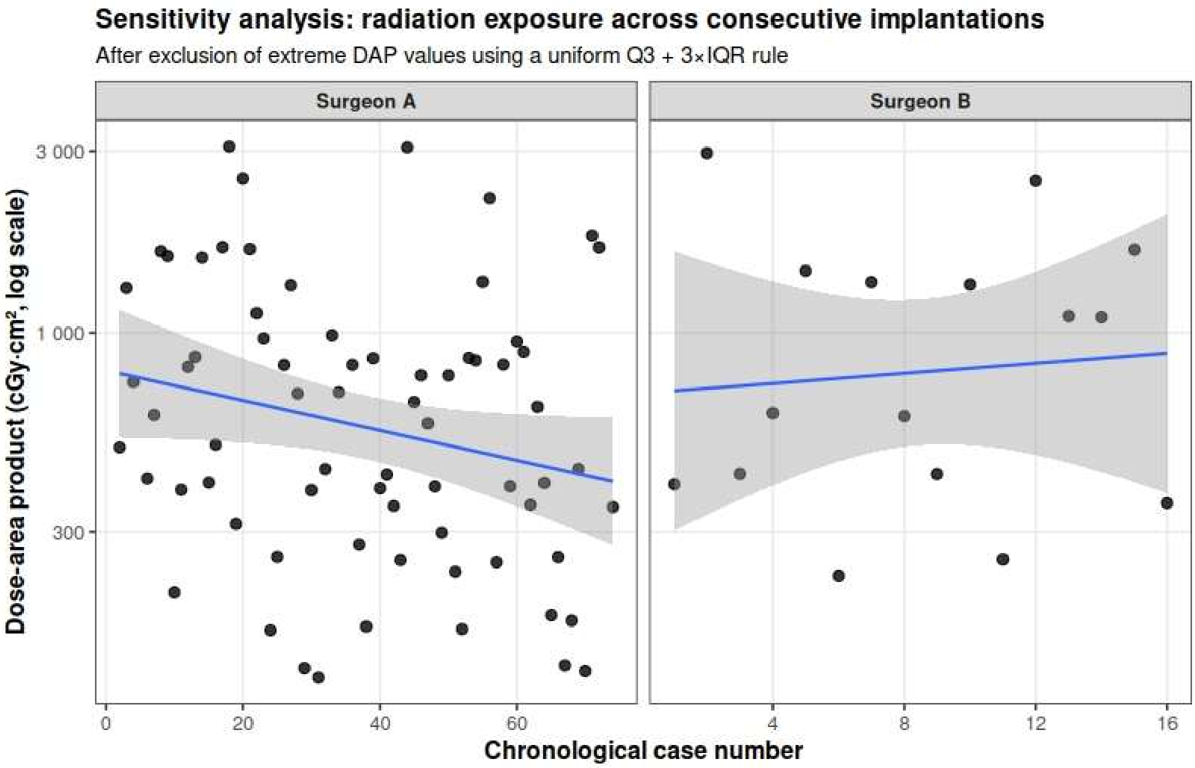

Supplementary Figure S2. Sensitivity CUSUM analysis after exclusion of extreme radiation values.

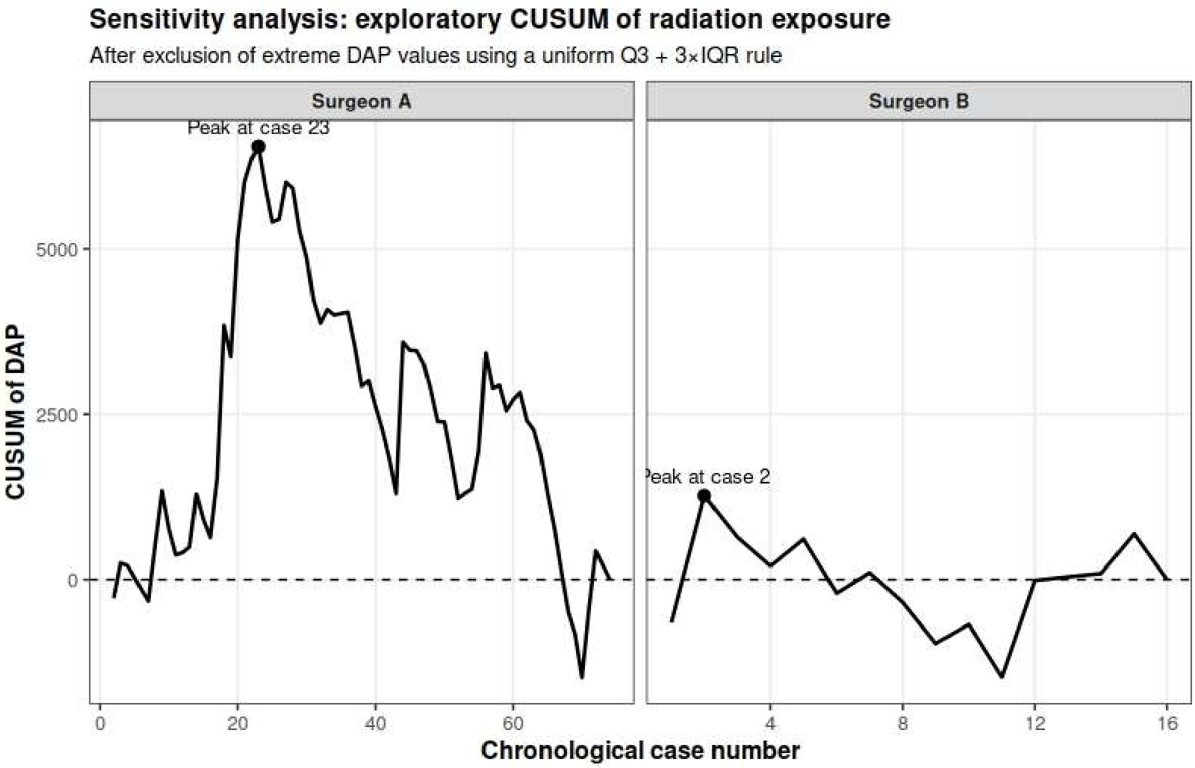

Supplementary Figure S3. Exploratory subgroup analysis by ECMELLA configuration in Surgeon A.

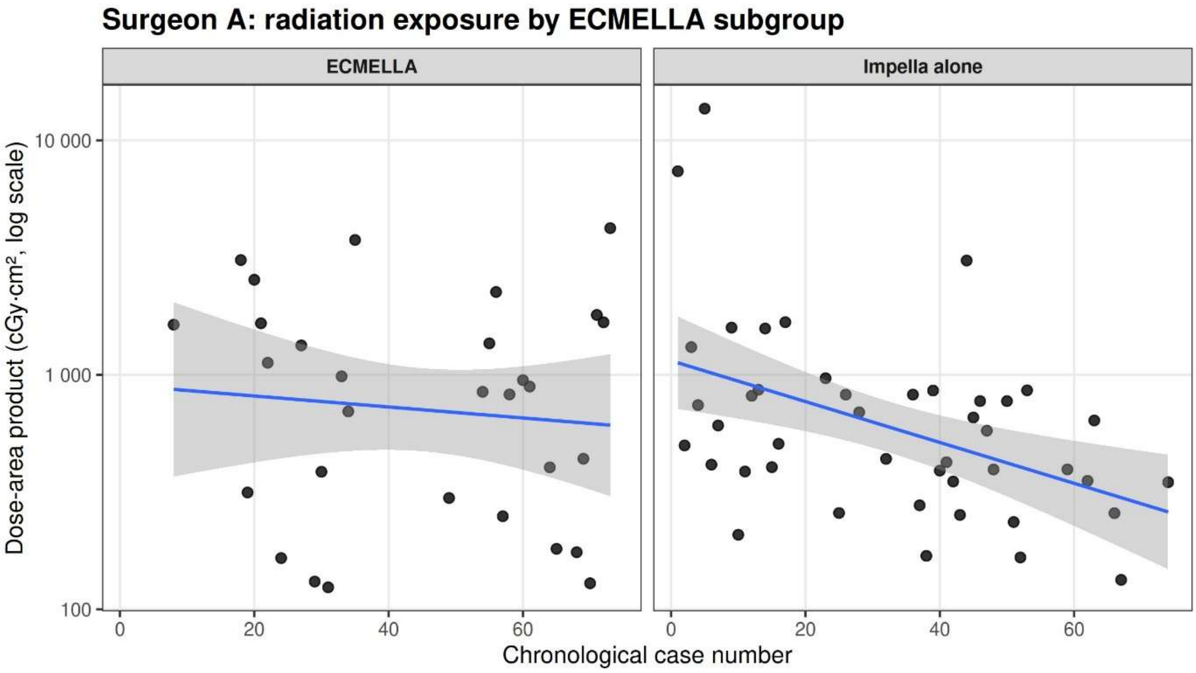

Supplementary Figure S4. Exploratory subgroup analysis by heart surgery status in Surgeon A.

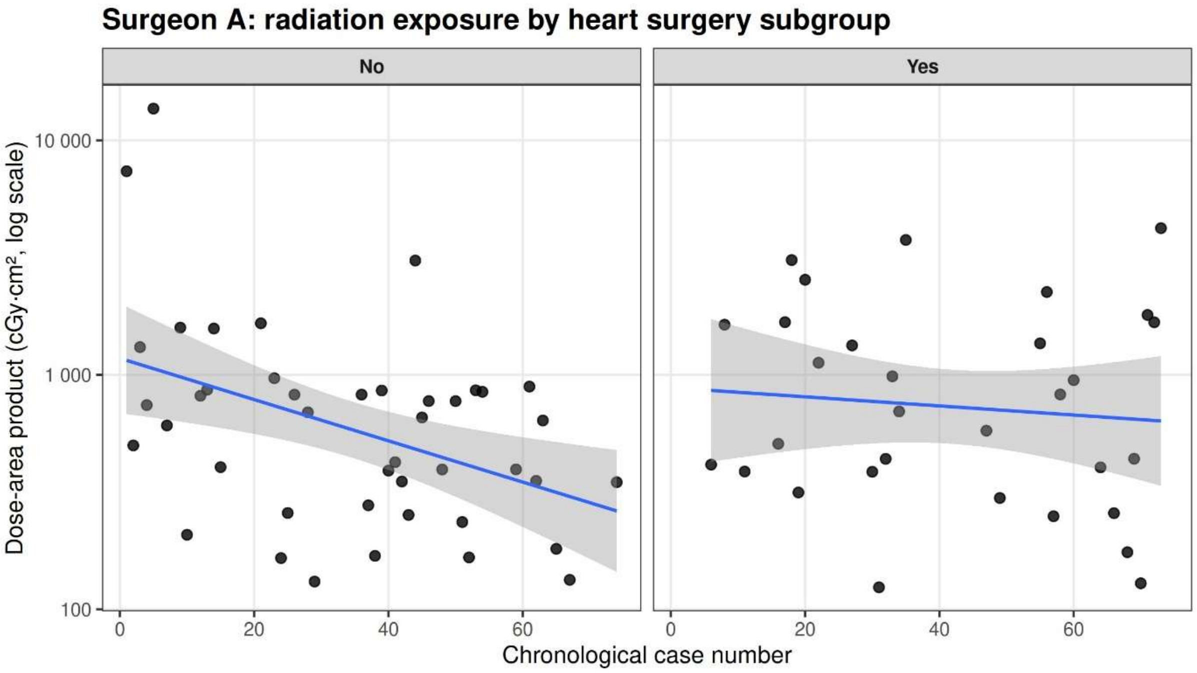

Supplementary Figure S5. Exploratory subgroup analysis by Impella platform in Surgeon A.

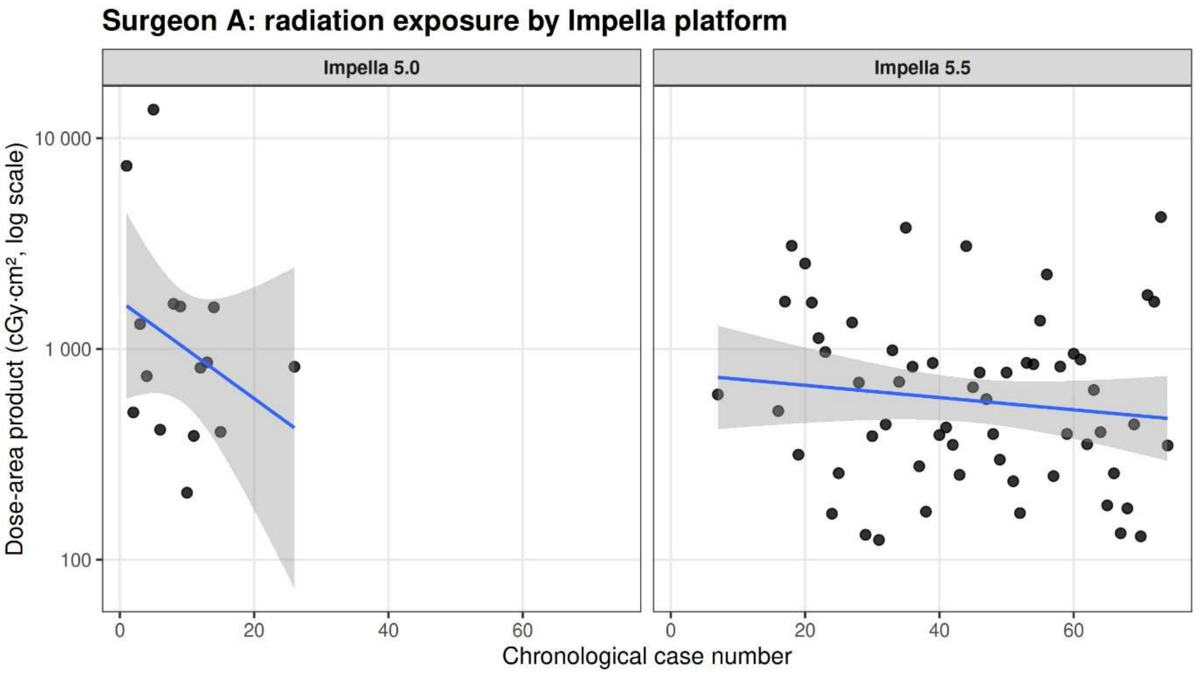

Supplementary Tables

Supplementary Table S1. Adjusted regression analyses

Supplementary Table S2. Exploratory subgroup results in Surgeon A

Supplementary Table S1. Adjusted regression analyses

Outcome	Variable	Estimate (95% CI)	P-value
log(DAP)	(Intercept)	6.669 (4.994 to 8.343)	<0.001
log(DAP)	case_number	-0.011 (-0.024 to 0.002)	0.084
log(DAP)	Surgeon B	-0.073 (-0.740 to 0.594)	0.828
log(DAP)	age	0.011 (-0.013 to 0.034)	0.369
log(DAP)	Ecmella configuration	-0.415 (-0.937 to 0.106)	0.013
log(DAP)	Concomitant procedures	0.129 (-0.374 to 0.632)	0.612
log(DAP)	Impella 5.5	-0.318 (-0.998 to 0.362)	0.355
Fluoroscopy time	(Intercept)	-0.786 (-20.430 to 18.858)	0.937
Fluoroscopy time	case_number	-0.018 (-0.171 to 0.134)	0.813
Fluoroscopy time	Surgeon B	1.277 (-6.547 to 9.101)	0.746
Fluoroscopy time	age	0.233 (-0.039 to 0.504)	0.092
Fluoroscopy time	Ecmella configuration	-0.274 (-6.387 to 5.840)	0.929
Fluoroscopy time	Concomitant procedures	0.435 (-5.465 to 6.335)	0.884
Fluoroscopy time	Impella 5.5	-3.084 (-11.058 to 4.891)	0.444

Supplementary Table S2. Exploratory subgroup results in Surgeon A

Descriptive analyses only

Subgroup domain	Subgroup	n	Age, years	Dose-area product, cGy·cm²	Fluoroscopy time, min
Configuration	ECMELLA	30	67.0 [60.0–70.8]	868.8 [302.5–1653.5]	6.7 [3.6–14.7]
	Impella alone	44	62.0 [56.0–70.2]	543.0 [352.7–832.0]	7.0 [4.3–12.8]
Concomitant procedures	No	43	62.0 [56.0–70.5]	638.9 [313.1–858.2]	7.0 [4.2–12.3]
	Yes	31	66.0 [59.5–70.5]	698.2 [386.5–1656.2]	6.1 [4.6–16.2]
Device type	Impella 5.0	15	62.0 [60.0–71.5]	823.2 [457.0–1583.0]	10.0 [5.0–15.5]
	Impella 5.5	59	64.0 [57.0–70.5]	578.0 [288.1–958.2]	6.3 [3.8–13.0]
